# Feasibility of a Commercially Available Virtual Reality System to Achieve Exercise Guidelines in Youth With Spina Bifida: Mixed Methods Case Study

**DOI:** 10.2196/20667

**Published:** 2020-09-03

**Authors:** Byron Lai, Drew Davis, Mai Narasaki-Jara, Betsy Hopson, Danielle Powell, Marissa Gowey, Brandon G Rocque, James H Rimmer

**Affiliations:** 1 Division of Pediatric Rehabilitation Medicine School of Medicine University of Alabama at Birmingham Birmingham, AL United States; 2 Department of Kinesiology California Polytechnic State University Pomona Pomona, CA United States; 3 Division of Pediatric Neurosurgery School of Medicine University of Alabama at Birmingham Birmingham, AL United States; 4 Department of Physical Medicine and Rehabilitation School of Medicine University of Alabama at Birmingham Birmingham, AL United States; 5 Division of Pediatric Gastroenterology, Hepatology, and Nutrition School of Medicine University of Alabama at Birmingham Birmingham, AL United States; 6 Dean's Office School of Health Professions University of Alabama at Birmingham Birmingham, AL United States

**Keywords:** physical activity, active video gaming, exergaming, disability, Oculus Quest

## Abstract

**Background:**

Access to physical activity among youth with spina bifida (SB) is much lower than it is for children without disability. Enjoyable home-based exercise programs are greatly needed.

**Objective:**

Our objective is to examine the feasibility of a virtual reality (VR) active video gaming system (ie, bundle of consumer-available equipment) to meet US physical activity guidelines in two youth with SB.

**Methods:**

Two youth with SB—a 12-year-old female and a 13-year-old male; both full-time wheelchair users—participated in a brief, 4-week exercise program using a popular VR head-mounted display: Oculus Quest (Facebook Technologies). The system included a Polar H10 (Polar Canada) Bluetooth heart rate monitor, a no-cost mobile phone app (VR Health Exercise Tracker [Virtual Reality Institute of Health and Exercise]), and 13 games. The intervention protocol was conducted entirely in the homes of the participants due to the coronavirus disease 2019 (COVID-19) pandemic. The VR system was shipped to participants and they were instructed to do their best to complete 60 minutes of moderate-intensity VR exercise per day. Exercise duration, intensity, and calories expended were objectively monitored and recorded during exercise using the heart rate monitor and a mobile app. Fatigue and depression were measured via self-report questionnaires at pre- and postintervention. Participants underwent a semistructured interview with research staff at postintervention.

**Results:**

Across the intervention period, the total average minutes of all exercise performed each week for participants 1 and 2 were 281 (SD 93) and 262 (SD 55) minutes, respectively. The total average minutes of moderate-intensity exercise performed per week for participants 1 and 2 were 184 (SD 103) (184/281, 65.4%) and 215 (SD 90) (215/262, 82.1%) minutes, respectively. One participant had a reduction in their depression score, using the Quality of Life in Neurological Disorders (Neuro-QoL) test, from baseline to postintervention, but no other changes were observed for fatigue and depression scores. Participants reported that the amount of exercise they completed was far higher than what was objectively recorded, due to usability issues with the chest-worn heart rate monitor. Participants noted that they were motivated to exercise due to the enjoyment of the games and VR headset as well as support from a caregiver.

**Conclusions:**

This study demonstrated that two youth with SB who used wheelchairs could use a VR system to independently and safely achieve exercise guidelines at home. Study findings identified a promising protocol for promoting exercise in this population and this warrants further examination in future studies with larger samples.

## Introduction

Spina bifida (SB) is a common birth defect that results in permanent mobility disability and affects ~170,000 people in the United States [1]. Approximately 37% percent of people with SB use a wheelchair or other assistive device as a primary means of mobility [2]. The importance of addressing obesity and physical inactivity (ie, insufficient levels of physical activity or its subset, exercise, to achieve health benefits) in this population cannot be understated. Approximately 37% of people with SB are obese [3], and they have higher fat mass (159%), lower cardiorespiratory endurance (32%-54% peak oxygen volume [VO_2_]), and lower muscular strength (58%-98%) compared to peers without SB [4]. Over time, these factors result in rapid physical deconditioning, placing them at risk for metabolic syndrome, cardiovascular disease, and type 2 diabetes [5]. There is a pressing need to identify effective approaches for living well with SB.

The transition from childhood to adulthood is a critical threshold for addressing the health needs of individuals with SB. Regular exercise is one of the most important health behaviors for reducing the risks associated with physical inactivity. Health professionals will typically instruct adolescents to try and achieve the exercise guidelines for children of 60 minutes per day of moderate-to-vigorous-intensity activity [6]. However, these recommendations are often unattainable for people who are physically inactive and there is evidence that adolescents with disabilities benefit from far lower volumes of physical activity [7]. Thus, health professionals will more commonly prescribe the adult exercise guidelines of 150 minutes of moderate-to-vigorous-intensity activity per week [6]. Youth who exercise regularly are more likely to live healthy, active lifestyles as adults and reduce their risk of chronic conditions.

For people with SB, several core concerns prompted the need for this pilot study:

1. Obesity rates for children with SB become alarmingly higher in adulthood [3].
2. Accessible and usable exercise opportunities within the community are scarce for youth with SB as they transition into adulthood [8].
3. Feelings of isolation and depression increase exponentially when transitioning into adulthood [9].
4. Social relationships, vocations or meaningful hobbies, and reduced dependence on caregivers are core developmental tasks during adolescence [10].
5. Wheelchair reliance increases as children with SB enter adolescence, which is partially due to excessive weight gain [2].

As reported in a systematic review [11], aerobic and strength interventions for youth with SB typically last 11 weeks, include two to three sessions per week, and total ~60 minutes of exercise per week. These encouraging, preliminary studies demonstrate potentially efficacious methods for improving aerobic capacity (ie, three studies, effect size range 0.74-1.40) and strength (ie, two studies, effect size range 0-0.59). However, these interventions included supervision from a therapist or research staff. To date, there is no program that can promote self-regulated exercise behavior among youth with SB, particularly individuals with mobility limitations who may experience difficulty with transportation to an on-site facility. Further research efforts are needed to identify accessible, enjoyable, and sustainable programs that can be easily engaged in by youth with SB.

Home-based exercise programs that deliver and monitor exercise prescriptions using telecommunication technology (ie, telehealth) are a desirable approach for people with disabilities who may not have convenient access to other means of exercise. The advantages of a telehealth approach over usual care include the following: increased social support, participant adherence, quality of care, cost-effectiveness, access to services, and, most notably, reduced trainer burden to allow easier dissemination of services [12]. Barriers to exercise can include lack of nearby accessible facilities, usable equipment, knowledgeable staff, and transportation [13,14]. For these reasons, exercise through telehealth is rapidly becoming the new norm during the coronavirus disease 2019 (COVID-19) pandemic.

Active video gaming (AVG) is an enjoyable method of home exercise that has the potential to engage youth with SB in long-term exercise behavior. AVG can be performed at a moderate intensity of exercise [15], making it a potentially effective method for improving health, function, and body composition. Virtual reality (VR)—computer and sensor technology that is used to create a simulated environment or experience—is an emerging area of AVG technology. In rehabilitation, VR technology is being used as an enjoyable method for managing pain and improving motor and executive function, fitness, movement quality, and spatial orientation and mobility [15-18]. However, only a handful of studies have investigated the influence of VR technology on promoting exercise behavior outside of a formal rehabilitation setting among youth with disabilities [15,16,19,20]. Furthermore, home-based studies have primarily incorporated less immersive forms of VR, including the Nintendo Wii and Xbox Kinect [15], but these technologies have been discontinued by game manufacturers.

Head-mounted display (HMD) technology is the latest trend in AVG. In May 2019, one of the largest investments in VR gaming by Facebook led to a pivotal advancement in making HMD more ubiquitous for consumers: the development of the Oculus Quest (Facebook Technologies). The Quest is the first VR headset of high visual quality (ie, up to 72 frames per second) that does not require any plug-in to a costly desktop gaming computer or game console. Higher frame rates provide a smoother visual display (aka, immersion) within a virtual environment [21], whereas lower frame rates can induce motion sickness and nausea [22]. The Quest has access to the latest top-rated VR fitness games and has online multiplayer capability that comes with free-to-play multiplayer cooperative and competitive recreational games. This makes the Quest the first off-the-shelf, all-in-one headset that allows health professionals to prescribe a high-quality immersive, enjoyable, and socially connected VR experience in the comfort of one’s home.

While there are a few studies that have demonstrated successful exercise responses in youth with SB [23,24], these studies have used nonstandalone HMDs: HTC Vibe and Oculus Rift. These devices had to be plugged in to a computer gaming system, which was stationed on-site at a school or rehabilitation clinic. To our knowledge, no study has demonstrated whether the Quest can be used for exercise training among children with disabilities, including SB. Moreover, we are not aware of any studies that promoted unsupervised exercise behavior that met or exceeded the duration component of the US exercise guidelines in youth with SB.

The case study in this paper tested the feasibility of using the Oculus Quest VR system to achieve exercise guidelines among two youth with SB who used a wheelchair as their primary means of mobility. Feasibility was evaluated via two *usability* metrics: *effectiveness* and *usefulness* [25]. The aims of this study were as follows:

1. Aim 1 (*effectiveness*): compare the minutes of moderate-intensity exercise achieved each week with exercise guidelines for children (60 min/day) and adults (150 min/week).
2. Aim 2 (*usefulness*): describe participants’ perceptions of factors that affected their ability to use the system to achieve the exercise guidelines.

The ancillary aim was to describe the potential treatment effects of the VR program on fatigue and depression.

## Methods

### Overview

The design was a mini-ethnographic case study for a convenience sample of two people [26,27]. A mini-ethnographic case design, also known as focused ethnography, is used for retrospectively developing a rich understanding of an individual or group response to a program or study [26]. This exploratory approach is smaller in scope and generally shorter in duration than a full-scale ethnographic approach, which embeds a researcher within a setting for a prolonged period of time to examine the lived experience through more pattern-focused analytical techniques (eg, grounded theory or thematic analysis) [26]. Ethnography is ideal for investigating the potential of innovative products or programs that are commonly used in medical and marketing research [26,28]. This design incorporated a nested mixed methods approach—QUANTITATIVE → qualitative [29]—for a comprehensive evaluation of feasibility. Specifically, the design included a qualitative postintervention interview within a primarily quantitative, feasibility preintervention-to-postintervention design. Quantitative and qualitative methods were combined within the Discussion section of this paper to provide an *expanded* evaluation of usability.

### Recruitment

This study purposefully selected one male and one female youth from the Spina Bifida Clinic at the Children’s Hospital of Alabama. Inclusion criteria were as follows: (1) less than 18 years of age, (2) used a wheelchair as a primary means of mobility, (3) physically inactive: in the previous 2 weeks, self-reported less than 150 minutes per week of moderate-to-vigorous-intensity exercise and no participation in a structured exercise program or regimen, (4) diagnosis of SB, (5) access to a mobile device: computer tablet or smartphone, and (6) Wi-Fi internet access in the home. Exclusion criteria were as follows: (1) owned a VR headset device or (2) had a health condition that prevented participation in moderate-intensity exercise. This study was conducted in accordance with case study guidelines set by the Institutional Review Board for Human Use at the University of Alabama at Birmingham (UAB). Written informed consent was obtained from participants prior to participation.

### Intervention Protocol

Participants were provided with a VR unit and asked to use the VR system to complete 60 minutes of moderate-intensity exercise per day, a guideline set by the US Department of Health and Human Services [6].

#### VR System

**Figure 1:**
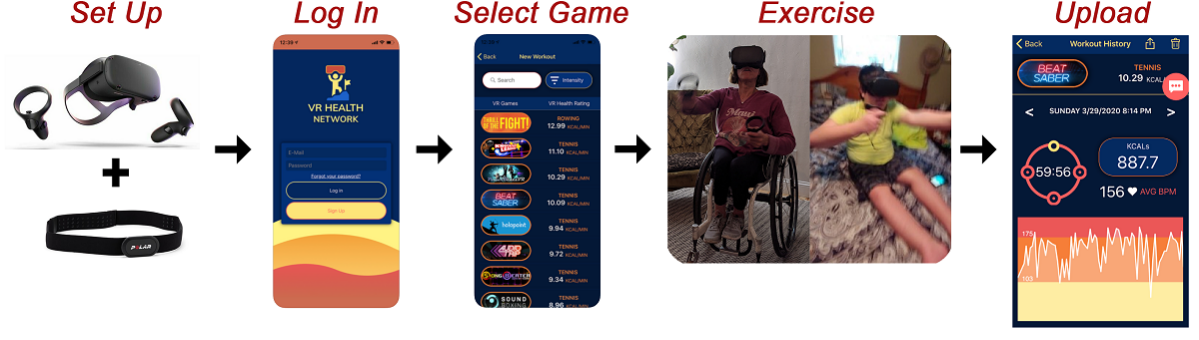
Exercise session procedures of the virtual reality (VR) system.

The VR system consisted of the following components (see Figure 1):

1. An Oculus Quest headset (64 GB) with two hand-held controllers.
2. A Polar H10 (Polar Canada) Bluetooth, chest strap, heart rate monitor.
3. A no-cost mobile phone app—VR Health Exercise Tracker (Virtual Reality Institute of Health and Exercise)—that records exercise duration and intensity.
4. A total of 13 games installed on the headset.

Prior to the start of the intervention, participants and one parent had a 30-minute videoconference call with the telecoach. The purpose of the call was to make sure they received the equipment and understood how to set up the system, reviewed the study procedures (ie, study objectives, the VR system, exercise prescription, and the postintervention interview), and briefly summarized the benefits of exercise. In accordance with the Supportive Accountability Theory [30], the goal of the brief communications—primarily the initial coaching call—was to develop a friendly relationship and bond with the participant and establish a social presence that provided a sense of accountability to enhance participant motivation and adherence to the study procedures.

In addition to the call, participants were provided with two pages of printed instructions. The instructions were presented in a bullet-list format that guided participants through the following setup procedures: (1) log in to the headset with an Oculus account that was created by the researcher; no identifying information from the participant was inputted or connected to an Oculus or Facebook account, (2) download the VR Health Exercise Tracker app on their phone, and (3) sync the Polar heart rate monitor to the VR Health Exercise Tracker app. The instructions also guided participants through the exercise session procedures: (1) log in to the VR Health Exercise Tracker app, (2) select a game, (3) turn on and equip the headset, (4) exercise with the selected game, and (5) after exercise, review and upload the exercise data into a cloud-based server.

During exercise, the VR Health Exercise Tracker app automatically recorded exercise data via Bluetooth from the Polar monitor. The Polar electrocardiographic chest strap monitors, including the H10, have over two decades of published studies demonstrating strong psychometric properties to support their use for measuring heart rate among both youth and adults [31-33]. VR Health Exercise Tracker records and displays in real time the following data: exercise intensity (heart rate), calories, and exercise duration (minutes). After each session, participants reviewed their exercise data and uploaded the data into a private folder within a cloud-based server (Google Drive). Based on the Tanaka equation [34], participant 1 (pseudonym: Johnny) was instructed to keep his exercise heart rate between 119 and 159 beats per minute; participant 2 (pseudonym: Sapphire) was instructed to keep her exercise heart rate between 120 and 160 beats per minute.

A telecoach (BL) reviewed and recorded exercise data obtained from the cloud-based server on a weekly basis. At the end of each week, the telecoach emailed the participants a progress report with graphs that displayed the total minutes and moderate-to-vigorous-intensity minutes of exercise completed. The reports included typed comments with positive verbal reinforcement of their exercise behavior, along with reminders to try their best to achieve the exercise prescription goals.

#### Intervention Games

The Quest headset that was sent to participants was preinstalled with 13 games. The games required dynamic, speed-driven movements of the arms that could be played in a seated position. The games included rhythmic movements to music as well as sport and recreation activities that elicited high energy expenditure (eg, dancing, boxing, and tennis), as determined by the Virtual Reality Institute of Health and Exercise [35]. Nine of the 13 fitness games were chosen by the telecoach because they could be performed while standing or sitting and had a high development quality. These core games were Beat Saber (movement to music), The Climb (mountain climbing), Racket Fury (table tennis), Thrill of the Fight (boxing), Sports Scramble (various sport and recreation activities), Half + Half, Rec Room, Dance Central, and a local multiplayer game that could be played with the family (Acron: Attack of the Squirrels!). Two of these games, Rec Room and Half + Half, were online group-based multiplayer games that could be downloaded at no cost from the Oculus Store. Acron: Attack of the Squirrels! is a cooperative and competitive game that matches up players (ie, squirrels) who use their personal mobile phones or tablet devices against the VR headset user (ie, tree); there is a maximum of 8 players. These three multiplayer games did not have a single-player game mode. Sports Scramble supported single-player and online multiplayer gameplay. Participants were allowed to choose the remaining four fitness games that they wanted installed on their headset. Chosen games included the following: Creed: Rise to Glory and BOXVR (two boxing games), Cloudlands 2 (golf), Racket: Nx (table tennis), AUDICA (movement to music), Synth Riders, and Moss. Participants were encouraged to test the suitability of each game for wheelchair exercise.

### Measures

The data collection was originally planned to be conducted on-site at a local fitness facility that had a human performance laboratory. In response to the societal impact of COVID-19, all data were collected remotely. Participants archived their data from each exercise session on the cloud-based server. Participants completed the study questionnaires before starting the 4-week intervention and a second time after they completed the 4-week intervention. Research staff sent the questionnaires to participants via email. Participants uploaded the completed questionnaires to the cloud-based server.

Fatigue and depression were measured via self-report, short-form questionnaires from the Neuro-QoL (Quality of Life in Neurological Disorders) Pediatric archive [36]. Each questionnaire had eight items that could be scored on a scale ranging from 1 (none of the time) to 5 (all of the time). Higher scores reflect higher levels of fatigue and depression. Neuro-QoL tests were systematically developed by a multisite collaboration and psychometrically evaluated among a large population-based sample [37]; they are currently being tested for further validation among more specific groups of people with neurological conditions (eg, traumatic brain injury, epilepsy, and muscular dystrophy) [38,39], albeit not yet among people with SB. Additional Neuro-QoL details and an extensive list of validation studies can be found elsewhere [36].

### Postintervention Interview

Participants underwent a semistructured interview with the researcher (BL) at the end of the program. The interview script is provided in Textbox 1. The interview included 10 overarching questions with several follow-up questions. The questions probed participants’ overall views of the program, likes and dislikes, experiences using the VR equipment for exercise during the program, barriers and facilitators to meeting the exercise prescription goals, preferences for the fitness games, and recommendations to enhance the protocol for a future trial. The interviews were conducted through videoconference. The participants were accompanied by their caregivers. The principal investigator (BL) had a background in adapted physical activity, along with several years of experience conducting qualitative interviews related to exercise and disability. Each interview lasted approximately 2 hours.

**Interview script.**

*Interview Briefing:*

“Good [morning, afternoon, evening]. This interview will include 10 questions, along with several follow-up questions. The questions will explore your perceptions of using the Oculus Quest headset to achieve exercise recommendations. This interview will be recorded so that we can later type and analyze the data for publication as a research study. This study is completely confidential and we will not include your name or any other identifying information. To keep it confidential, please don’t say any names of people or places. Your participation in the study will not affect any services or relationships you have with people at the University or Hospital.”

“Before we start, please give me a fun and fake name to call you so that I do not say your real name during the interview.”

Participant Pseudonym:_________________

*Audio Recording Started (TURN ON AUDIO RECORDING and go to next page):

“I have now started the audio recording. Once again, your participation in this study is totally voluntary and your verbal consent over the phone now indicates that we have gone over the study details and that we have permission from you and one of your parents to participate in this phone call interview.”

“Just so I can get a picture of you, I’m going to ask you a few questions”:

1. How old are you?

2. What ethnicity are you?

3. How tall are you?

4. How much do you weigh?

5. Do you wear orthotics or use ambulatory aides? If so, what kind?

6. Do you use a wheelchair? If so, what kind?

7. Generally, do you live in a rural area or urban city area?

“Okay, before we get started, please be open and honest with your responses. Please say the first things that come to your head: what you think and what you feel. There are no right or wrong answers. Please just tell your story. Even though we are on the telephone, don’t be afraid to keep talking. I will try to be listening more than talking during the interview.”

*Interview Questions (Prompts: “Could you tell me more about that?,” “How did that make you feel?,” “Do you have anything else you’d like to add about that?”):

1. As an icebreaker, tell me about yourself. What do you like to do for fun or in your free time?

2. Okay, please take me through what a typical week/day is for you.

3. What does exercise mean to you? (images, words, activities, anyone ever talk to you about it?, advice)

“Okay, this next question will be a bit different. For this question, we will discuss your story about your experiences with the virtual reality headset, the Oculus Quest. We will talk about your thoughts of exercise starting from when you first started the program all the way to the end of the month.”

4. Okay, so let’s start. Before you started the program, like when I first called you, how did you feel about starting an exercise program using the virtual reality headset?

*Follow-up Questions:*

i. How confident were you that you could exercise with the technology?

ii. Before you started the program, did you exercise? (how often?)

iii. How did you feel about exercise?

iv. Were you feeling motivated or energized to do the exercises?

5. Okay, so in the first week of the program (show data). What did you think about exercising with the headset?

*Follow-up Questions:*

i. How confident were you that you could exercise with the technology?

ii. Were there any problems you had with using the technology for exercise?

iii. Were there any things you really liked?

iv. Were there any things you really didn’t like?

v. Was it difficult to do 60 minutes of exercise each day?

vi. Was it difficult to do 60 minutes of MODERATE-intensity exercise each day?

vii. Were you feeling motivated or energized to do the exercises?

viii. Were there any issues with the technology?

ix. Were there any things you think we could have done in this first week to make the experience better?

6. Okay, so now let’s talk about the second week of the program (show data). What did you think about exercising with the headset during the second week?

*Same Follow-up Questions as 5*

7. Okay, so now let’s talk about the third week of the program (show data). What did you think about exercising with the headset during the third week?

*Same Follow-up Questions as 5*

8. Okay, so now let’s talk about the fourth week of the program (show data). What did you think about exercising with the headset during this last week?

*Same Follow-up Questions as 5*

9. Okay, next question; we are planning to give the same program you went through to other kids with spina bifida around your age. Are there any thoughts you have that could make the program better for other kids?

10. Okay, video game time! What was your favorite game to play?

*Follow-up Questions:*

i. What did you like about each game? Did you find it easy to get your heart rate to the moderate zone from that game?

ii. For each game you mentioned, what are some tips or strategies you can recommend for other kids? (Go through each game they can recall).

iii. What was your least favorite game? (Follow up with why).

11. Do you have anything else you would like to add?

### Analysis

All quantitative outcomes (ie, objectively recorded exercise data and self-reported questionnaire data) were descriptively reported. To address Aim 1, the average minutes of moderate-to-vigorous-intensity exercise performed each week were compared with both the youth and adult guidelines: ≥420 minutes of moderate-to-vigorous-intensity exercise per week (ie, >60 minutes per day) was classified as *excellent* (youth guidelines); ≥150 minutes of moderate-intensity exercise per week was classified as *sufficiently active* (adult guidelines); and <150 minutes of moderate-intensity exercise per week was classified as *insufficiently active* (adult guidelines). Neuro-QoL depression and fatigue scores at baseline and postintervention were summed and converted to T scores [40].

The qualitative component utilized an explanatory narrative approach [41] to explain the potential usefulness of the program and expand interpretations of the quantitative data. Interview data were transcribed and double-checked for accuracy. The results were reported in narrative format, underpinned by an interpretivism paradigm. The specific philosophical assumptions that underpinned the qualitative study methods were critical realism (ontological perspective) [42] and interpretivism (epistemological perspective) [43]. In other words, the researcher team acknowledged that youth with SB perceived a reality when reporting their responses, and the recollection of this reality or experience was recreated by the interaction between the youth and the interviewer, as well as the interpretation of the data.

## Results

### Overview

Characteristics of the two participants are presented in Table 1. The quantitative exercise data are reported below, followed by an explanatory narrative of each case. There were no adverse events reported by participants throughout the program. The participants completed the program in April 2020, a period when states within the United States were under a *social distancing* and *shelter-in-place* order.

**Table 1:** Case characteristics.

Characteristic	Case 1: Johnny	Case 2: Sapphire
Age (years)	13	12
Sex	Male	Female
Race	Caucasian	Caucasian
Height (feet, inches)	5, 3	5, 0
Weight (pounds)	174	132
Body mass index (kg/m^2^)	30.8	25.8

### Quantitative Exercise Data

**Figure 2:**
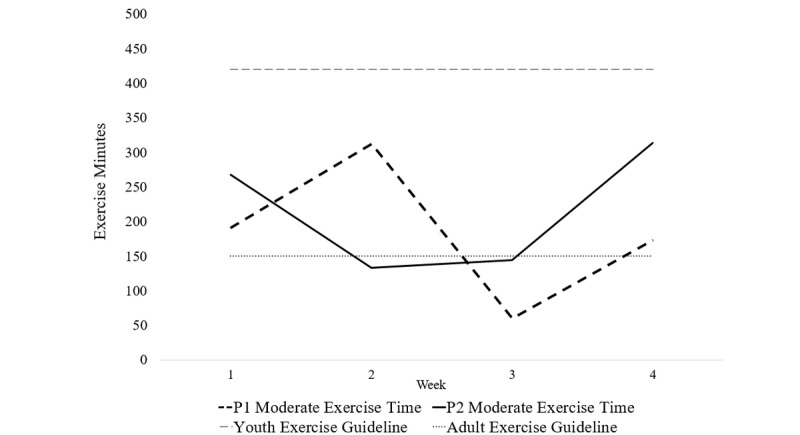
Moderate-intensity exercise minutes completed by each participant. P1: participant 1, Johnny; P2: participant 2, Sapphire.

The total average (SD) minutes of exercise performed each week for case 1 (Johnny) and case 2 (Sapphire) were 281 (SD 93) and 262 (SD 55) minutes, respectively. The total average minutes of moderate-to-vigorous-intensity exercise per week for cases 1 and 2 were 184 (SD 103) and 215 (SD 90) minutes, respectively. Accordingly, both case participants were classified as *sufficiently active*. These values were below the exercise guidelines for children, but exceeded exercise guidelines for adults (see Figure 2). On average, participants spent 71.1% (SD 12%) of their time exercising at a moderate intensity. Weekly exercise data are reported in Table 2.

**Table 2:** Weekly exercise data.

Measure		Participant 1: Johnny	Participant 2: Sapphire
**Moderate-intensity exercise (minutes), n/N (%)**			
	Week 1	191/364 (52.5)	268/304 (88.2)
	Week 2	312/312 (100)	133/212 (62.7)
	Week 3	60/147 (40.8)	144/216 (66.7)
	Week 4	173/299 (57.9)	314/314 (100)
Number of days exercised		24	15
Heart rate per day of exercise (beats per minute), mean (SD)		127 (17)	127 (15)
Caloric expenditure per day of exercise (kcal), mean (SD)		496 (215)	441 (145)
Total number of exercise sessions		29	29
Total number of moderate-intensity exercise sessions		21	25

Both participants reported average levels of depression at baseline (case 1: Neuro-QoL score of 12, T score=47.9; case 2: Neuro-QoL score of 13, T score=48.8). Case 1 reported an improvement in depression from baseline to postintervention (change in T score of 11.4) that surpassed a minimal detectable change of 9.6 at case level [40]. Case 2 reported no change in depression score at postintervention. Fatigue scores at baseline were low and did not change at postintervention (case 1: Neuro-QoL baseline score of 10, T score=36.5, and postintervention score of 12, T score=39.5; case 2: baseline Neuro-QoL score of 15, T score=42.8, and postintervention Neuro-QoL score of 14, T score=41.8).

### Explanatory Qualitative Cases

#### Case Summary

The case summary is shown in the items below:

1. Participants engaged in no exercise prior to the intervention.
2. Before COVID-19, participants engaged in no exercise outside of a school setting, and they were not included in many physical education activities.
3. Participants were hesitant to join the study but became confident in their ability to exercise throughout the program.
4. Participants reported a high level of enjoyment and immersion with the VR headset and games, which motivated them to exercise.
5. Caregivers facilitated exercise behavior and helped participants adhere to the monitoring procedures.
6. Participants reported that they engaged in far more exercise than what was recorded objectively, due to issues with the chest-worn heart rate monitor.
7. Three preferences for active games were identified: opponent-driven fighting games, multiplayer games, and rhythmic movement-to-music games.
8. Participants reported several minor usability issues: low battery life, headset and heart rate monitor discomfort, troubles with online multiplayer play, and some games requiring modifications for accessibility.

#### Case 1: Johnny

##### Overview

Johnny was a 13-year-old Caucasian male that lived in a suburban area in Alabama, USA. Johnny used a wheelchair as a primary means of mobility and used an ankle-foot orthosis (AFO) when walking short distances. Prior to the COVID-19-related *shelter-in-place* order and the study, Johnny engaged in no exercise behavior outside of school physical education classes. Watching and attending sporting events, particularly football, was one of Johnny’s favorite hobbies. Yet, he was unable to engage in most sports and activities with his peers during physical education class. The activities in his class were not fully adapted or modified for a wheelchair user. Thus, Johnny spent much of his physical education time isolated from participation on the sidelines. Once COVID-19 imposed quarantine restrictions, he engaged in no exercise behavior. Most of his free time was spent watching sports on television or playing nonactive video games.

At the start of the program, Johnny reported that he was not confident that he could complete 60 minutes of moderate-intensity exercise in a day. He felt intimidated by these instructions, and stated that the duration felt long and he could not remember the last time he completed 60 minutes of any exercise in one day. These feelings were maintained until he opened the box with the headset that was sent to his home. Immediately upon viewing the headset, Johnny was extremely excited by the quality of the technology and quickly set up the system and played the games. The first day he received the headset, he used it for over 3 hours. After using the VR headset for 1 week, he realized that exercising for 60 minutes a day at a moderate intensity could easily be achieved and did so in his first day of using the headset. He was very excited and proud of his ability to exercise with the headset and would consistently show his family some of the VR experiences and demonstrate his physical prowess during gameplay. His mother reported that she would have to limit his daily use of the headset so as not to interfere with mealtime and schoolwork. His mother also reported that he spent approximately three times as much time using the headset than what was recorded through the VR system: approximately 4 hours each day. Headset use mostly included active games, but some of this time included passive device use, such as a free-to-download VR roller coaster ride or “every once in a while” watching shows on Netflix. Johnny exercised with the headset in a wheelchair, chair, or bed or on the floor.

##### Facilitators

Johnny reported a high level of enjoyment while playing the fitness games, which was attributed to the immersion or “felt real” factor provided by the system and the fun games: “I loved it. All of it. I loved it.” Johnny stated that his favorite games were those where he had to overcome an opponent—Creed: Rise to Glory and Thrill of the Fight—and the rhythmic movement-to-music game, Beat Saber. Johnny did not spend much of his time playing online multiplayer games, but one particularly enjoyable multiplayer game was Acron: Attack of the Squirrels! Johnny played this game with his entire family, which created some memorable experiences. Johnny reported that VR games were more enjoyable than typical console games and that he liked being able to play the game while moving his arms. His mother noted that he was so engaged with some of the games that his family members had to be careful around him: “My mom was sitting beside him and he knocked her glasses off and I was like, ‘You have to watch out. He cannot see you’ (laughs).” Prior to the VR program, Johnny noted that he spent 3-4 hours playing console games, such as Fortnite, or sports games each day, but he quit playing these games after using the VR headset. He stated that he preferred the VR games because “I like being able to play the game while I’m moving my arms.” It is important to mention that Johnny reported that his motivation to exercise was primarily due to the enjoyment of the games and headset, but his mother highly motivated him to adhere to the exercise data collection protocol. Johnny’s mother would consistently remind him to wear his heart rate monitor to record the data from each session. Johnny’s mother was the person exporting the exercise data to a cloud-based server.

Notably, Johnny and his mother reported that he seemed to have lost some weight based on his facial features and some other areas of his body. Johnny’s mother also mentioned a change in shirt size from extra-large to large from pre- to postintervention. Both Johnny and his mother were elated at being able to see an observable change in such a short period. Johnny was particularly thrilled that he was able to lose weight, which was an important goal of his, while doing activities that were so enjoyable that he was unaware he was exercising. He also reported that the calories expended after each session provided him with a sense of accomplishment. By accomplishing these feats and realizing that he was capable of performing a high volume of exercise, Johnny felt more motivated to engage in general exercise after the program. No objective measurement of weight was obtained, due to difficulties with obtaining body weight measurements while using a wheelchair. The last weight measurement that the family obtained was during a visit to their physician prior to the COVID-19 quarantine. Johnny’s mother appreciated the opportunity to engage her son in the study, since the typical outdoor activities they engaged in during summer (eg, camp and parks) were unavailable due to the COVID-19 quarantine.

##### Barriers and Usability Issues

Johnny did much less exercise in weeks 2 and 3 than in weeks 1 and 4. Johnny and his mother noted that this was because he was traveling with his father in a conventional sleeper truck. Johnny brought the Quest with him while accompanying his father and would play in the truck while his father drove or before bed. Also, during week 3, Johnny did not feel well due to a flare-up of his allergies and this reduced his playing time. Johnny reported that he did not like to wear and equip the heart rate monitor before exercise. He noted that it felt uncomfortable and he would occasionally unfasten the strap during long bouts of exercise. For these reasons, much of the exercise he did was not recorded with the VR system. Johnny recommended that his heart rate be recorded from a wrist-worn device, such as his Apple Watch. Notably, Johnny mentioned that the battery life of the headset was too short in duration (~2 hours), which required him to consistently charge the headset after using it each day. Johnny reported that he tried the multiplayer games but did not engage in them regularly. He and his mother reported that there did not appear to be many other players when joining a game lobby. Unlike Xbox Live, they did not notice a big community of players. Instead, he spent more time playing local multiplayer games with his entire family. Family members played the games—Acron: Attack of the Squirrels! and Rec Room—using their mobile phones while Johnny played on the headset, and this provided them with several cherished opportunities to bond during the COVID-19 quarantine.

#### Case 2: Sapphire

##### Overview

Sapphire was a 12-year-old Caucasian female living in a suburban area in Alabama, USA. Sapphire used a wheelchair as a primary means of mobility and used an AFO when walking short distances. Similar to Johnny, Sapphire engaged in no exercise behavior outside of her school’s physical education classes prior to the COVID-19 quarantine. At school, she was not included in most sports and activities with her peers during physical education class. Physical education classes included group activities, which Sapphire did not participate in: “I just watch the kids. I don’t do anything.” One day each week was devoted to exercises, and Sapphire felt more included within these activities. However, at a previous school she did have an adapted physical education teacher. The teacher developed a personalized program for Sapphire and modified activities to include her in the activities with peers. During the COVID-19 quarantine, Sapphire engaged in no exercise behavior and spent most of her free time watching television or using her phone. However, Sapphire and her mother reported that she had a high volume of schoolwork in the form of homework and virtual class sessions via videoconferencing. Since the family shared one laptop for work and school, they operated around a strict schedule. This meant that VR exercise had to be performed early in the morning before school or later in the evening.

Before Sapphire began the program, she was worried that she would not be able to complete the prescribed 60 minutes. The amount of time was perceived as too long and intimidating. Sapphire’s mom encouraged her not to worry and to think more about playing than exercising. When the headset arrived at their home and Sapphire saw it, she was extremely excited and could not wait to try it out. She was surprised to realize that she completed 78 minutes of exercise in her first session. She said it was “very fun” and she could not wait to do it again. By the end of the study, it was very easy for her to complete 60 minutes of moderate exercise. She often wanted to play the VR games longer than prescribed and to get back on the headset later in the day. When exercising, Sapphire would sit in a wheelchair or chair.

##### Facilitators

Sapphire expressed a high level of enjoyment with the program. Similar to Johnny, Sapphire acknowledged that the virtual world “felt real” and was quick to report her excitement while playing certain games. Sapphire was so encapsulated by the virtual environment that she pleasantly described her experience and knowledge of four friends she made in a game, Dance Central, which the interviewer did not realize were in-game characters, not actual online players, until further clarification. However, the games that Sapphire preferred were slightly different than the opponent-driven fighting games preferred by Johnny. Sapphire preferred multiplayer games—AUDICA, Acron: Attack of the Squirrels!, Sports Scramble, and Rec Room—as well as rhythmic movement-to-music games: AUDICA and BOXVR. Sapphire specifically mentioned her enjoyment playing multiplayer games online, such as Rec Room, with other users and, most notably, at home with the family: Acron: Attack of the Squirrels! Sapphire consistently desired to play with her family and asked her father to play as soon as he returned home from work:

You ask him when he walks straight through the front door (laughs).

Mother

So? I want to play with him!

Sapphire

When asked how she was able to achieve so much exercise time, she stated that in the first week of the program she completed multiple 30-minute bouts as opposed to one 60-minute session. It became easier for her to complete 60-minute exercise bouts during the second week of the program. Another factor that motivated her to exercise was being able to view the calories she expended each day within the VR Health Exercise Tracker app.

Similar to Johnny, Sapphire’s mom played a major role in the program. As stated by Sapphire’s mom, “I helped her put the heart rate monitor on. I would get the VR off of her, charge it, hand it to her. She would put it on. I would help her secure the controllers to her wrist, so she doesn’t accidentally throw them.”

Sapphire’s mom also helped schedule Sapphire’s exercise time. This assistance was instrumental during the first 2 weeks of the program. However, during the latter 2 weeks of the program, Sapphire appeared internally motivated to complete her exercises. As stated by her mom, “The last week she [Sapphire] was dead set. No matter what, she would say we were going to make an hour or more each time.”

##### Barriers and Usability Issues

During weeks 2 and 3, Sapphire’s mom reported that there was a transition to a full-time school load delivered through the internet due to the COVID-19 quarantine. This required them to adjust their already strict schedule, which contributed to a lower amount of exercise. Sapphire reported that there was a learning curve to using the VR headset and games. It took 2 days of exercise before she and her mother figured out how to equip the headset comfortably over her glasses and adjust the headset appropriately to avoid pressure on the front of the face, which would cause uncomfortable temporary imprints on her forehead.

Although Sapphire reported that some games were perfectly suitable for playing in a wheelchair, other games—Dance Central and Racket Fury—required lowering the player height in either the game or Oculus Quest settings for optimal play. Default settings for the player height of these games caused many of the activities to be out of physical reach for Sapphire. Additionally, Dance Central instructed players to move their lower extremities, which Sapphire could not follow. Some games (eg, tennis) took up a considerable amount of floor space, and Sapphire and her mom had to coordinate play time in the living and dining rooms to avoid conflict with family activities (eg, television and meals).

When Sapphire would play a multiplayer game online, such as Rec Room, some other players would speak foul language. Sapphire was instructed by her mom to change game rooms until she was able to play the games with others who did not use offensive language. Similar to Johnny, Sapphire’s mom stated that the heart rate monitor was sometimes not worn when playing, indicating that she spent more time exercising than what was recorded. The heart rate monitor had difficulties in syncing with the phone. Similar to Johnny, Sapphire also reported that the VR headset would have to be charged frequently due to the low battery life. She was using the headset for such long durations of play that the battery life (ie, 2.5 hours) could be expended within 1 or 2 days.

## Discussion

### Principal Results

This study was the first to objectively measure whether physically inactive youth with SB could engage in health-enhancing exercise behavior over a typical 1-month intervention period at home, using a state-of-the-art VR headset (ie, Oculus Quest). The study was implemented during a *shelter-in-place* order by the state due to the COVID-19 pandemic. Both participants reported that they engaged in no exercise behavior since COVID-19-related school closures, which was 2-3 weeks prior to starting the study. Yet, objective data indicated that both participants could use the headset to satisfy and exceed adult exercise guidelines of 150 minutes of moderate-intensity exercise across a 1-month period.

Although the levels of exercise obtained by participants were lower than 420 minutes of moderate-intensity exercise per week, which are part of the exercise guidelines for children [6], satisfying the adult exercise guidelines is an important finding for several reasons. First, adult exercise guidelines are widely prescribed as the minimum criteria for receiving health benefits from exercise. Second, the ability to maintain this level of exercise behavior will be important for living a healthy, active lifestyle as youth transition into adulthood. Third, benefits to health have been observed from even far lower levels of exercise behavior among youth with disabilities [7,11]. Fourth, levels of physical activity were higher than those reported in published exercise interventions for youth with SB [11] and other groups with physical disabilities [7]. There is a need to build on these findings and examine the effects of VR on critical health and function outcomes in this population.

The findings from the qualitative interviews helped explain the quantitative exercise data. First, participants reported that the amount of exercise they completed was heavily facilitated by the immersive and enjoyable movement that was involved with the VR games. Second, caregivers were identified as an agent that facilitated exercise behavior. Caregivers managed their child’s exercise time and had a vital role in physically assisting them in setting up, adjusting, and using the VR system. Caregiver support appeared most influential for ensuring that participants equipped the heart rate monitor during exercise. Third, due to usability issues identified with the chest-worn heart rate monitor, the actual amount of exercise completed by each youth was likely far higher than what was objectively recorded by the VR system. In fact, both caregivers had to limit the participants from overusing the headset in order to not interfere with school-related work. Last, while participants did achieve an encouraging level of exercise behavior throughout the program, they identified minor usability issues that had to be resolved for an optimal gameplay experience.

The findings from this case study identified a promising protocol that can be used to engage youth with SB in health-enhancing levels of exercise behavior at home. A strength of this protocol was that youth were intrinsically motivated to play the VR games and, thus, did not need extensive behavioral coaching from research staff. Each participant was provided with one 20-minute coaching call at the start of the program and brief weekly reports. This required no more than a total of 1 hour and 40 minutes of time from research staff throughout the 1-month intervention. Thus, the protocol has minimal burden on research staff, making it highly useful for implementing in larger efficacy or effectiveness trials.

A second strength of this protocol was that participants were able to achieve a respectable volume of moderate-intensity exercise each week while playing games at a *normal* or *hard* level of difficulty. This is an important finding since the provided games typically had much higher levels of difficulty (eg, an easy, normal, hard, expert, and expert + difficulty setting). Given that higher game difficulties require faster and more dynamic movements and a higher level of executive function, participants still had substantial room for growth using the same intervention games. It took the researcher (BL), who had a background in professional-level gaming, approximately 3 months of daily play to master the hardest difficulty level of the provided games. The longevity of this type of program for youth with SB needs to be evaluated.

### Future Recommendations

Lessons can be learned from this case study to inform the design of future efficacy or effectiveness trials. A bullet-point list of recommendations for future trials is shown below (see Textbox 2).

#### Caregiver

The most notable lesson this study was the importance of caregiver support. Caregivers were not provided with direct instructions to assist the participants with the VR exercise program. Yet, caregiver support naturally emerged as a critical facilitator of exercise behavior, and this should be the standard moving forward in a larger trial. Caregivers have substantial on influence their child’s adherence to home-based programs [44,45]. In this study, caregivers were highly motivated to support their child in performing the VR games, which will not always be the case in a larger trial or, perhaps, a time when families are not quarantined to the home due to COVID-19. Future studies should include behavioral coaching strategies that can accompany VR gaming for not only the child participant but also for the caregiver.

#### Convenient Methods of Monitoring

A second notable lesson for future VR exercise trials is the need for a more convenient method of monitoring exercise data. The chest-worn heart rate monitor was inconvenient for the two participants and was identified as a barrier to adhering to the monitoring protocol. Caregivers often had to support the children in this process. This ultimately resulted in a lower objectively recorded volume of exercise than was performed. The monitor for this study was chosen for its compatibility with the VR Health Exercise Tracker app and for the evidence supporting its accuracy and precision. To enhance the likelihood that youth will comply with the monitoring procedures, future studies should consider incorporating more convenient monitoring devices, such as those worn on the wrist or arm. However, the cost and accuracy of wrist-worn devices vary considerably (67%-92% agreement with the Polar H7 monitor) [46]. Researchers and health professionals will have to weigh convenience, accuracy, and cost when choosing a monitoring device. Regarding exercise data, participants seemed to highly value the resultant calorie data over data for heart rate after each exercise session. Thus, an ideal monitoring protocol might include a valid and reliable wrist-worn device that can sync with an easy-to-use mobile phone app or the headset itself to monitor heart rate, calories, and exercise time.

#### Intervention Instructions

A third important lesson learned from this study was that participants and caregivers should be provided with instructions on how to properly set up, equip, and adjust the headset. Every time the Quest is turned on, the device requires the user to set and draw the maximum boundaries for the play area as a measure of safety. If an individual moves outside of these bounds, they no longer see the game through their headset and instead view their surrounding area. Participants can exercise through the headset in either a stationary space (~5 feet in diameter) or *room-scale* space (maximum 25 × 25 feet). A strength of the Quest is that it includes an *inside-out* tracking system (ie, built-in motion sensors within the headset that track arm and leg use) to allow the participant to easily change play locations, whereas most other HMDs of similar visual quality require external wall-mounted cameras and, thus, one designated play location. Nevertheless, families should be informed of these space requirements to avoid conflicts with their daily activities or interruptions to the exercise session. Notably, participants will need specific instructions or accessories (eg, counterweights, cushions, or elastic headband support) for adjusting the headset to the face of a child. Prolonged exercise bouts with the headset sometimes caused temporary, uncomfortable facial imprints. Included within the calibration of the headset is the ability to set the player’s height. People who use wheelchairs must be sure to set the player height to eye-level height while sitting, or change the height level if a specific game does not have built-in features to accommodate a nonstanding player (eg, Dance Central). Otherwise, games will be out of arm’s reach for the user.

#### Game Characteristics

This study also provided valuable insight on game characteristics that may be useful for developers and future interventionists. An encouraging finding was that most of the provided games for the Quest could be played using default game settings with only the participants’ arms. The chosen games included most of the nonviolent active games that were available on Quest at the time of the study. Fortunately, most games for the Quest are based on dynamic arm movements instead of leg movements, which is a generally accessible feature for wheelchair users. Only a couple of games encouraged heavy use of the lower limbs or specific adjustments to default game settings (ie, lowering the player height) to be played successfully (ie, Dance Central and Racket: Nx). These games might require specific instructions for adaptation or modifications for accessibility if they are to be included in another trial.

The participants had slightly different preferences for game genres. Johnny spent most of his time playing single-player, action-oriented, and opponent-driven fighting games, whereas Sapphire generally preferred more cooperative and competitive multiplayer experiences. Providing participants with a variety of games within these genres might be important for promoting play. One notable exception was the rhythmic movement-to-music games, which were enjoyed by both participants, making this genre a potentially important one for development, modification, or inclusion for male and female youth with SB.

Although the youth were provided with both single-player and multiplayer games, the time spent playing multiplayer games appeared low, or participants did not choose to wear the monitoring system while playing these games. One participant perceived the online multiplayer community to be small. The other participant was exposed to inappropriate language by other users. Multiplayer games foster competition, cooperation, and friendships and, thus, have the potential to be more engaging over a long period of time. Further strategies for promoting and monitoring multiplayer gameplay are warranted.

Finally, the perceived realism of using the Quest appeared to be an important factor in participants’ perceived enjoyment of, and likely their adherence to, the VR experience. This finding agrees with previous studies, whereby enjoyment was linked with VR immersion [47]. Participants reported that the experience “felt” real, not simply “looked” real, which implies that both the optical and proprioceptive systems were adequately immersed. This is likely due to the Quest having six degrees of freedom, a feature that is only included in the more sophisticated VR devices; a high-quality visual display; and no cable plug-in or external wires. Researchers and developers should aim to maintain or improve on this *realism* factor when developing future exercise-related VR trials or technology.

**Intervention recommendations for future trials.**

- Caregivers should assist their child in using the hardware and software, when necessary, and encourage them to participate in the required amount of daily exercise.
- Exercise data, including caloric expenditure, should be objectively recorded by wrist- or arm-worn devices.
- Instructions should be provided to participants for setting up and adjusting the headset; video instruction could be especially helpful.
- Games that are usable for wheelchair users include the following: Beat Saber, AUDICA, Thrill of the Fight, Creed: Rise to Glory, Rec Room, Acron: Attack of the Squirrels!, Sports Scramble, and BOXVR.
- Strategies that encourage multiplayer gameplay should be developed and tested.

### Limitations

Inherent within a case study design, the study findings should be interpreted with caution and will need to be confirmed in a larger efficacy trial. This study explored potential feasibility and usability issues and, thus, could not provide a more confirmatory result of acceptable or nonacceptable feasibility and usability, which could have been done through a single-subject research design. Similarly, we were unable to determine the impact or importance of the brief telecoach interactions with participants on exercise behavior. Another limitation was the lack of objective health and fitness laboratory measures, which would have provided estimates for treatment effects in a larger trial. Due to the COVID-19 quarantine, on-site testing was restricted at the university where the study was conducted. The COVID-19 quarantine may also have affected the level of engagement of both the participants and parents, as the study was implemented during a *shelter-in-place* order. In addition, the cost of the VR equipment may limit broad use for some populations.

### Conclusions

This study tested the feasibility of using the latest state-of-the-art technology in consumer-available VR gaming among two youth with SB who used wheelchairs. Participants used the headset to safely achieve exercise guidelines, and this protocol holds promise for promoting physical activity on a larger scale among youth with SB. We identified several modifications to the VR exercise protocol and knowledge gaps that can be pursued in a future efficacy trial.

## Abbreviations

AFO: ankle-foot orthosis
AVG: active video gaming
COVID-19: coronavirus disease 2019
HMD: head-mounted display
Neuro-QoL: Quality of Life in Neurological Disorders
SB: spina bifida
UAB: University of Alabama at Birmingham
VO_2_: oxygen volume
VR: virtual reality
